# Neonatal Outcomes Following Elective Induction of Labor at 39 Weeks: A Systematic Review

**DOI:** 10.7759/cureus.88277

**Published:** 2025-07-19

**Authors:** Abeer Ahmed, Hanady ME M Osman, Nada Salaheldin Suliman Ali, Salma Mohammed Elbashir Salih Ahmed, Habab Mohamed Musa Mohamed, Hoyam Mutasim Yousif Bakheit, Alkhansaa Mahmod Mohamed Alhag

**Affiliations:** 1 Obstetrics and Gynecology, Royal Infirmary Hospital of Edinburgh, Edinburgh, GBR; 2 Quality and Patient Safety, Najran Armed Forces Hospital, Ministry of Defense Health Services, Najran, SAU; 3 Emergency Department, Burjeel Hospital, Abu Dhabi, ARE; 4 Pediatrics, Liwa Extended Health Center, Liwa, OMN; 5 Pediatrics, King Fahad Armed Forces Hospital, Jeddah, SAU; 6 Emergency Department, Sabt Alalayah Hospital, Sabt Alalayah, SAU; 7 Neonatal Intensive Care Unit, King Saud University, Riyadh, SAU

**Keywords:** 39 weeks gestation, cesarean delivery, elective induction of labor, neonatal outcomes, perinatal mortality, systematic review

## Abstract

Elective induction of labor (eIOL) at 39 weeks of gestation has gained prominence in obstetric practice, yet its impact on neonatal outcomes remains debated. This systematic review aimed to synthesize evidence on neonatal outcomes following eIOL at 39 weeks compared to expectant management, addressing critical knowledge gaps to inform clinical decision-making. Following PRISMA guidelines, a comprehensive search of PubMed, Scopus, Web of Science, and Embase identified 16 eligible studies. Inclusion criteria focused on low-risk pregnancies undergoing eIOL at 39 weeks, with neonatal outcomes as primary endpoints. Risk of bias was assessed using the Newcastle-Ottawa Scale for cohort studies and Cochrane RoB 2 for the RCT. Narrative synthesis was performed due to heterogeneity. Key findings demonstrated that eIOL at 39 weeks was associated with reduced cesarean delivery rates and lower perinatal mortality, without significant increases in adverse neonatal outcomes. However, subgroup analyses revealed variability: obese women benefited from reduced macrosomia and NICU admissions, while women with prior cesareans faced higher failed TOLAC rates. The RCT confirmed lower cesarean rates but no reduction in composite neonatal morbidity. eIOL at 39 weeks is a safe and effective strategy for reducing cesarean deliveries and perinatal mortality in low-risk populations, though benefits vary by subgroup. Shared decision-making, tailored to maternal characteristics, is essential. Future research should prioritize RCTs in high-risk populations and long-term neonatal follow-up.

## Introduction and background

Elective induction of labor (eIOL) has become an increasingly common obstetric intervention in modern maternity care [1]. Traditionally, induction of labor was reserved for maternal or fetal indications such as hypertensive disorders, post-term pregnancy, or fetal growth restriction. However, in recent decades, there has been a shift in clinical practice towards offering elective induction at term, particularly at 39 weeks of gestation, even in the absence of medical indications [2]. This shift is driven by emerging evidence suggesting potential maternal and neonatal benefits, as well as changes in obstetric policies aiming to optimize perinatal outcomes and resource utilization [3].

Globally, the prevalence of labor induction has steadily increased, with significant variation across countries depending on local guidelines, resource availability, and obstetric culture [4]. For example, in the United States, the ARRIVE trial [5] conducted by the Maternal-Fetal Medicine Units Network in 2018 provided robust evidence that elective induction at 39 weeks in low-risk nulliparous women was associated with a reduction in cesarean delivery rates compared to expectant management, without an increase in adverse neonatal outcomes. This landmark trial has influenced clinical recommendations and has prompted widespread discussions about the balance between maternal benefits, neonatal safety, and healthcare system implications of elective induction at 39 weeks.

Neonatal outcomes associated with elective induction at 39 weeks remain a subject of active investigation [6]. While term delivery is generally considered optimal for neonatal health, concerns have been raised about potential risks associated with non-spontaneous initiation of labor. These concerns include respiratory morbidity, NICU admissions, feeding difficulties, and transient tachypnea of the newborn, as labor induction may not fully replicate the physiological and hormonal processes of spontaneous labor onset [7]. Conversely, elective induction at 39 weeks may reduce risks associated with advancing gestation, such as macrosomia, meconium aspiration syndrome, and stillbirth, thus potentially improving overall neonatal outcomes [8].

The complexity of this clinical decision-making is further underscored by differences in study designs, population characteristics, induction protocols, and outcome definitions across existing literature. Observational studies and randomized controlled trials have reported varying results regarding neonatal morbidity, making it challenging for clinicians to provide clear counseling to pregnant women considering elective induction at this gestational age. Additionally, systematic synthesis of neonatal outcomes across different healthcare settings is necessary to inform evidence-based guidelines and to identify any subpopulations that may derive particular benefit or harm from this practice.

Given these considerations, it is imperative to systematically evaluate the available evidence to understand the full spectrum of neonatal outcomes associated with elective induction of labor at 39 weeks compared to expectant management. This systematic review aims to address this critical knowledge gap by synthesizing findings from recent studies to provide a comprehensive assessment of neonatal risks and benefits, thereby informing clinical practice, patient counseling, and future research directions.

## Review

Methodology

This systematic review was conducted in accordance with the Preferred Reporting Items for Systematic Reviews and Meta-Analyses (PRISMA) guidelines [9].

Eligibility Criteria

Studies were included if they met the following criteria: (1) Population: Pregnant women undergoing eIOL at 39 weeks gestation compared to expectant management; (2) Intervention: Elective induction of labor at 39 weeks with no medical indication; (3) Comparator: Expectant management (defined as spontaneous labor or induction at a later gestational age); (4) Outcomes: Neonatal outcomes (e.g., perinatal mortality, Neonatal intensive care unit (NICU) admission, Apgar scores, respiratory morbidity) and maternal outcomes (e.g., cesarean delivery, peripartum complications); (5) Study Design: RCTs, prospective and retrospective cohort studies. Case reports, editorials, reviews, and studies focusing solely on maternal outcomes without neonatal data were excluded.

Search Strategy

A comprehensive literature search was performed across multiple databases, including PubMed, Scopus, Web of Science, and Embase. The search strategy combined Medical Subject Headings (MeSH) terms and free-text keywords related to "elective induction of labor," "39 weeks," "neonatal outcomes," and "expectant management." The full search syntax for each database is provided in the supplementary table in the Appendix. Additionally, manual searches of reference lists from relevant reviews and included studies were conducted to identify additional eligible articles.

Study Selection and Data Collection Process

Two independent reviewers from the list of authors (HMMO and NSSA) screened titles and abstracts for eligibility, followed by full-text assessment of potentially relevant studies. Discrepancies were resolved through discussion or consultation with a third reviewer (HMMM). Data extraction was performed using a standardized form, capturing study characteristics (author, year, country, design, sample size), population demographics, intervention details, comparator definitions, and outcome measures. Authors of studies with incomplete data were contacted for clarification where necessary.

Risk of Bias Assessment

The methodological quality of included studies was evaluated using the Newcastle-Ottawa Scale (NOS) [10] for cohort studies and the Cochrane Risk of Bias Tool (RoB 2) [11] for RCTs. The NOS assessed selection (representativeness, exposure ascertainment), comparability (confounder adjustment), and outcome (follow-up, blinding) domains, with scores of 7-9 indicating low risk, 4-6 moderate risk, and ≤3 high risk. The RoB 2 tool evaluated randomization, deviations, missing data, outcome measurement, and selective reporting. Disagreements in bias assessment were resolved by consensus.

Data Synthesis and Analysis

Due to heterogeneity in study designs, populations, and outcome definitions, a narrative synthesis was conducted rather than a meta-analysis. Outcomes were categorized thematically and summarized in a table.

Results

Study Selection Process

**Figure 1:**
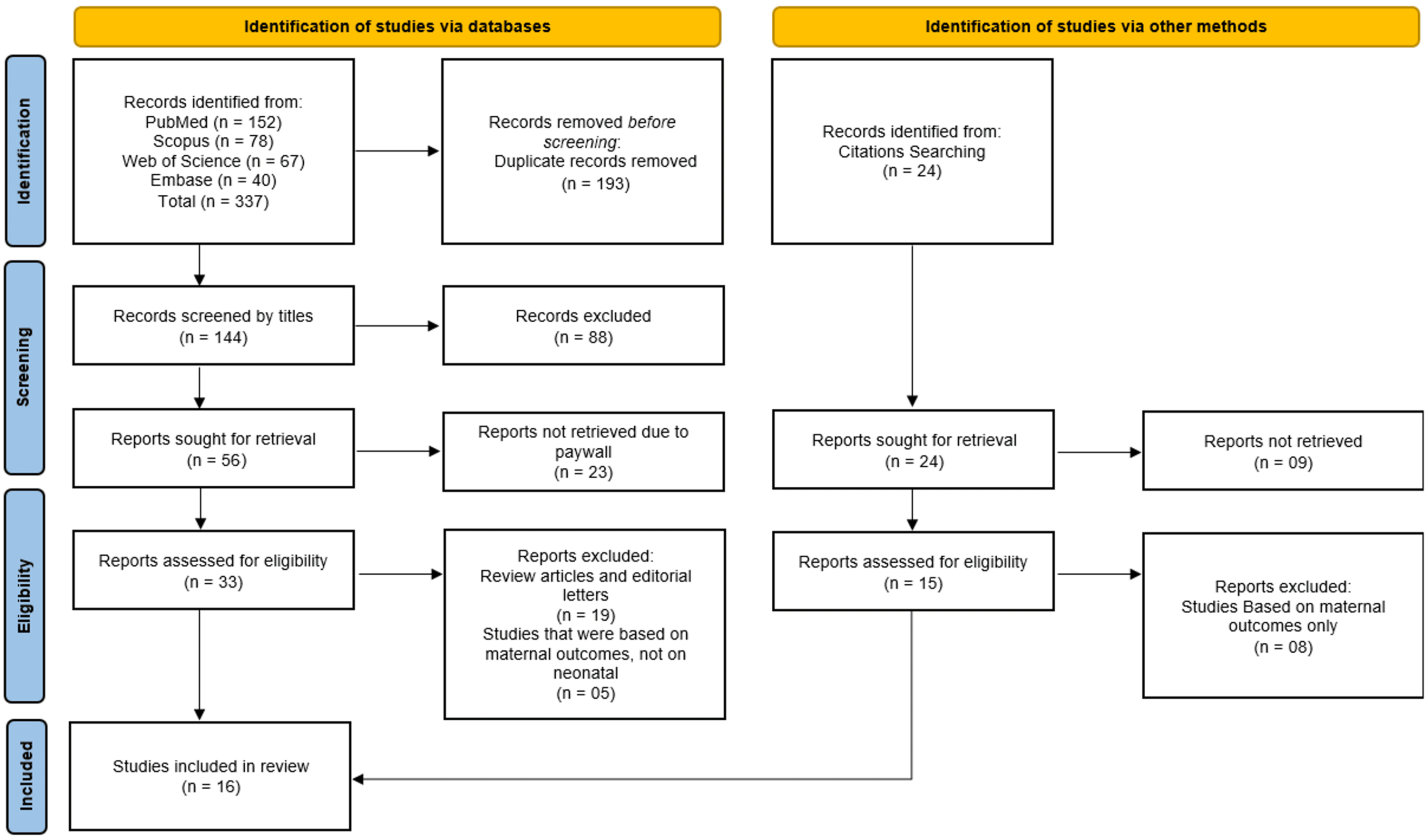
PRISMA Flowchart Illustrating Study Selection Process

The systematic review began with 337 records identified from database searches (PubMed: 152, Scopus: 78, Web of Science: 67, Embase: 40) and 24 additional citations from other sources. After removing 193 duplicate records, 144 studies were screened by title, excluding 88 irrelevant records. Following abstract and full-text screening, 56 reports were sought for retrieval, with 23 unavailable due to paywalls. Of these, 33 full-text articles were assessed for eligibility, excluding 19 review articles/editorials and 8 studies focused solely on maternal outcomes. Ultimately, 16 studies [5, 12-26] met the inclusion criteria and were included in the systematic review (Figure 1).

Characteristics of Included Studies

The systematic review included 16 studies [5, 12-26] examining neonatal outcomes following eIOL at 39 weeks compared to expectant management. The studies were conducted across diverse geographical regions, including Egypt [12], Australia [13], Austria [14], Scotland [15], and the USA [5, 16-26]. Study designs varied, with prospective and retrospective cohort studies predominating, alongside one multicenter randomized controlled trial [5]. Sample sizes ranged from 68 [12] to over 1.2 million [15], encompassing diverse populations such as nulliparous women, obese women, and those with prior cesarean deliveries. Key characteristics of the included studies are summarized in Table 1.

**Table 1:** Key Characteristics and Findings of the Included Studies

Author (Year)	Country	Study Design	Setting	Sample Size (n)	Population Characteristics	Intervention	Comparator	Neonatal Outcomes Measured	Main Findings
Grobman et al., [5] (2018)	USA	Multicenter randomized controlled trial	Multiple centers across USA (multicenter trial)	6106 (3062 induction, 3044 expectant management)	Low-risk nulliparous women at 38+0 to 38+6 weeks gestation	Elective induction of labor at 39+0 to 39+4 weeks	Expectant management	Composite of perinatal death or severe neonatal complications	Primary outcome occurred in 4.3% (induction) vs. 5.4% (expectant management), RR 0.80 (95% CI 0.64–1.00); cesarean rate significantly lower in induction group (18.6% vs. 22.2%, RR 0.84, 95% CI 0.76–0.93)
Elhoseny et al., [12] (2025)	Egypt	Prospective cohort study	Suez Canal University Hospital	68	Nulliparous women ≥39 weeks gestation	Elective induction at 39 weeks with vaginal misoprostol (50 µg)	Expectant management until spontaneous labor or 42 weeks	Fetal distress, NICU admission, Apgar scores	Induction reduced CS rate (8.8% vs 29.4%) with no significant difference in neonatal outcomes
Crawford et al., [13] (2025)	Australia	Retrospective cohort study	Queensland, population-based	472,520	Low-risk, singleton pregnancies between 2000 and 2021	Planned birth at 39+0–39+6 weeks gestation (including induction of labour [n=39,697] and scheduled caesarean section [n=57,741])	Expectant management	Perinatal mortality (antepartum or intrapartum stillbirth and neonatal death), severe neonatal neurological morbidity, severe neonatal non-neurological morbidity, severe maternal outcome, maternal-infant separation, perineal trauma, shoulder dystocia, caesarean birth	Planned birth at 39 weeks was associated with significantly lower odds of perinatal mortality (52% reduction), antepartum stillbirth (62% reduction), intrapartum stillbirth (84% reduction), severe neonatal neurological morbidity (54% reduction), severe non-neurological morbidity (35% reduction), and severe maternal outcomes (5% reduction).
Zenzmaier et al., [14] (2021)	Austria	Retrospective cohort study	Hospital births	447,066	Singleton term and post-term births (37–41 weeks gestation) between 2008–2016	Non-medically indicated IOL at each week from 37–41	Expectant management defined as “next week or beyond” or “at next week”	Cesarean delivery rates	Non-medically indicated IOL at 39 weeks resulted in comparable cesarean rates to expectant management defined as “next week or beyond” but higher rates compared with “at next week”. The definition of expectant management significantly impacts interpretation of IOL outcomes.
Stock et al., [15] (2012)	Scotland	Retrospective cohort	Consultant and midwife-led obstetric units (1981-2007)	1,271,549	Singleton pregnancies ≥37 weeks gestation	Elective induction of labor at 37-41 weeks with no medical indication	Expectant management (continuation to spontaneous labor, induction, or C-section at later gestation)	Extended perinatal mortality, admission to neonatal/special care baby unit	Elective induction at each gestation from 37-41 weeks was associated with decreased odds of perinatal mortality but increased neonatal unit admissions before 41 weeks.
Souter et al., [16] (2019)	USA	Retrospective cohort study	21 hospitals in the Northwest US	55,694 births (4002 elective inductions at ≥39 weeks; 51,692 non-elective births at 39+0–42+6 weeks)	Singleton cephalic hospital births at 39+0–42+6 weeks gestation; excluded prior cesarean, fetal anomalies, diabetes, hypertension, stillbirth	Elective induction of labor at ≥39 weeks gestation	Expectant management (ongoing pregnancies in same gestational week)	Macrosomia, 5-minute Apgar <7, resuscitation at delivery, intubation, respiratory complications, NICU admission	In nulliparous women, elective induction at 39 weeks reduced cesarean birth risk and pregnancy-related hypertension but increased operative vaginal birth; in multiparous women, similar cesarean rates and reduced pregnancy-related hypertension; no significant increase in adverse neonatal outcomes.
Sinkey et al., [17] (2019)	USA	Retrospective cohort study	Single center	3,703	Low-risk multiparous women delivering nonanomalous singletons between 39–42 weeks (2014–2018)	Elective induction of labor at 39 weeks (39 0/7 to 39 4/7)	Expectant management	Perinatal composite: death, neonatal respiratory support, 5-min Apgar ≤3, shoulder dystocia; NICU admissions	Elective induction at 39 weeks was associated with decreased perinatal composite morbidity (4.0% vs 7.1%; aOR 0.57) and lower cesarean rate (5.1% vs 6.6%; aOR 0.60); no difference in NICU admissions or other maternal outcomes
Park et al., [18] (2022)	USA	Retrospective cohort analysis	CDC National Center for Health Statistics, Division of Vital Statistics (national database)	50,136	Singleton, non-anomalous pregnancies with one previous cesarean delivery, between Jan 2015-Dec 2017	Elective induction of labor at 39 weeks (n=9,381)	Expectant management until 42 weeks (including induction, augmentation, or spontaneous labor between 40-42 weeks)	Low 5-minute Apgar score, NICU admission, prolonged neonatal ventilation, neonatal seizure, perinatal/neonatal death	Elective induction at 39 weeks decreased risk of low 5-min Apgar score (0.31% vs 0.47%, aRR:0.66), intra-amniotic infection, and blood transfusion, but increased cesarean delivery risk (49% vs 27.6%). No differences in other neonatal outcomes.
Palatnik and Kominiarek, [19] (2020)	USA	Retrospective cohort study	Consortium on Safe Labor (multicenter data from 2002-2008)	17,087 (7,298 nulliparous; 9,789 parous women)	Obese women (BMI ≥30 kg/m²), singleton gestations at ≥39 weeks, without medical comorbidities, excluding medically indicated inductions	Elective induction of labor at 39, 40, or 41 weeks	Expectant management	Macrosomia	Elective induction at 39 weeks was associated with lower cesarean delivery rates in both nulliparous and parous women compared to expectant management. Macrosomia was reduced in nulliparous women induced at 40 weeks and in parous women induced at 39 and 40 weeks.
Lee et al., [20] (2016)	USA	Retrospective cohort	Deliveries in California (2007)	74,725	Obese women with term, singleton, vertex, nonanomalous deliveries	Elective IOL at 37, 38, 39, or 40 weeks	Expectant management at corresponding gestational ages	Macrosomia, shoulder dystocia, brachial plexus injury, respiratory distress syndrome	eIOL at 38-40 weeks was associated with lower odds of macrosomia. No differences in odds of shoulder dystocia, brachial plexus injury, or respiratory distress syndrome. eIOL reduced cesarean delivery risk, particularly among multiparous women.
Lappen et al., [21] (2015)	USA	Secondary analysis of a large obstetric cohort study (Consortium on Safe Labor)	Multi-center (hospitals participating in Consortium on Safe Labor)	6,033	Women with term (≥37 weeks) singleton gestation and one prior cesarean delivery attempting TOLAC	Induction of labor (at 37-40 weeks)	Expectant management (at corresponding gestational weeks)	Composite neonatal morbidity (5-min Apgar <5, cord pH <7.0, asphyxia, hypoxic ischemic encephalopathy, neonatal death), neonatal ICU admission	Induction increased risk of neonatal ICU admission at 37 weeks (adjusted OR 2.51) but was not associated with increased overall neonatal morbidity.
Gibson et al., [22] (2014)	USA	Retrospective cross-sectional study	12 US institutions (19 hospitals)	131,243 deliveries (13,242 electively induced)	Healthy women with viable, vertex singleton pregnancies at 37-41 weeks of gestation	Elective induction of labor at 37-41 weeks	Expectant management	Major, minor, and respiratory neonatal morbidity composites	Elective induction at ≥38 weeks was associated with lower neonatal morbidity (e.g. at 39 weeks adjusted OR 0.75 (95% CI, 0.61–0.92)) compared to expectant management
Pickens et al., [23] (2018)	USA (California)	Retrospective cohort	Hospital-based	165,975	Singleton, cephalic, nonanomalous deliveries to obese women (both nulliparous and parous), 39–41 weeks of gestation	Elective induction of labor at 39–41 weeks	Expectant management	NICU admission	Elective induction at 39 and 40 weeks was associated with reduced NICU admissions (nulliparous: 7.9% vs 10.1%, aOR 0.79; parous: 5.3% vs 7.4%, aOR 0.75) and reduced maternal morbidity and cesarean rates compared to expectant management.
Darney et al., [24] (2013)	USA (California)	Retrospective cohort study	Statewide hospital discharge and vital statistics database (California, 2006)	362,154	Women without prior cesarean delivery; vertex, nonanomalous, singleton deliveries at term (37–40 weeks)	Elective induction of labor at 37–40 weeks	Expectant management	Perinatal death, NICU admission, respiratory distress, shoulder dystocia, hyperbilirubinemia, macrosomia	Elective induction associated with decreased odds of cesarean delivery across all gestational ages. No increased odds of perinatal death, NICU admission, respiratory distress, shoulder dystocia (except increased at 39 weeks), or macrosomia. Increased hyperbilirubinemia at 37 & 38 weeks.
Cheng et al., [25] (2012)	USA	Retrospective cohort study	Deliveries in the USA (2003)	132,112	Singleton births of macrosomic neonates (4000 ± 125 g) to low-risk nulliparous women at ≥39 weeks of gestation	Induction of labor at 39 weeks	Expectant management (delivery at 40, 41, or 42 weeks by induction or spontaneous labor)	5-minute Apgar scores, neonatal injury, method of delivery (C-section rate)	Induction at 39 weeks was associated with lower caesarean delivery rates compared to delivery at later gestational ages; similar trends observed at 40 and 41 weeks.
Bailit et al., [26] (2015)	USA	Retrospective cohort study	25 hospitals across the US	31,169	Nulliparous women with vertex nonanomalous singleton gestations, low-risk, delivered between 38 0/7 and 41 6/7 weeks	Nonmedically indicated elective induction at each week (38-41 weeks)	Expectant management during that same week	Neonatal complications (composite), peripartum infections, third- and fourth-degree lacerations, admission-to-delivery time, cesarean delivery	At 39 weeks, nonmedically indicated induction was associated with lower maternal and neonatal morbidity compared to expectant management; less frequent peripartum infections and severe lacerations were observed; induction was linked with longer admission-to-delivery time and higher cesarean rates at 38 and 40 weeks but not specified at 39 weeks

Neonatal Morbidity and Mortality

Elective induction at 39 weeks was associated with reduced perinatal mortality in several studies. Crawford et al. [13] reported a 52% reduction in perinatal mortality (aOR 0.48, 95% CI 0.30-0.76) and significant declines in antepartum (62% reduction) and intrapartum stillbirth (84% reduction). Similarly, Stock et al. [15] found a lower odds of perinatal mortality with eIOL (adjusted OR 0.39, 99% CI 0.24-0.63). However, Grobman et al. [5] observed no statistically significant reduction in a composite of perinatal death or severe neonatal complications (RR 0.80, 95% CI 0.64-1.00), though the cesarean rate was significantly lower in the induction group.

Neonatal morbidity outcomes were mixed. Elective induction at 39 weeks was linked to lower rates of severe neonatal neurological morbidity (54% reduction) and non-neurological morbidity (35% reduction) [13]. Conversely, Stock et al. [15] reported increased neonatal unit admissions (adjusted OR 1.14, 99% CI 1.09-1.20), particularly before 41 weeks. Other studies, including Souter et al. [16] and Sinkey et al. [17], found no significant differences in adverse neonatal outcomes such as low Apgar scores, respiratory complications, or NICU admissions.

Cesarean Delivery and Maternal Outcomes

A consistent finding across studies was the reduction in cesarean delivery rates with eIOL at 39 weeks. Elhoseny et al. [12] reported a cesarean rate of 8.8% in the induction group versus 29.4% with expectant management. Similarly, Gibson et al. [22] noted a lower cesarean rate with eIOL (adjusted OR 0.47, 95% CI 0.38-0.57), and Grobman et al. [5] observed an 18.6% cesarean rate in the induction group compared to 22.2% with expectant management (RR 0.84, 95% CI 0.76-0.93). However, Park et al. [18] found an increased cesarean risk with eIOL (aRR 1.72, 95% CI 1.68-1.77), particularly among women with prior cesarean deliveries.

Maternal outcomes were generally favorable with eIOL. Bailit et al. [26] reported fewer peripartum infections (OR 0.66, 95% CI 0.49-0.90) and severe lacerations (OR 0.60, 95% CI 0.42-0.86), while Crawford et al. [13] noted a modest reduction in severe maternal outcomes (5% reduction).

Specific Populations

In obese women, eIOL at 39 weeks was associated with reduced macrosomia [19, 20] and lower NICU admission rates [23]. For women with prior cesarean deliveries, Lappen et al. [21] found increased failed TOLAC rates with induction (adjusted OR 2.16, 95% CI 1.76-2.67) but no increase in neonatal morbidity.

Summary of Neonatal Outcomes

Table 2 synthesizes neonatal outcomes across studies. Key findings include reduced perinatal mortality, lower cesarean rates, and no significant increase in adverse neonatal outcomes with eIOL at 39 weeks, though some studies reported higher neonatal unit admissions or cesarean risks in specific subgroups.

**Table 2:** Neonatal Outcomes Following Elective Induction at 39 Weeks Compared to Expectant Management

Author (Year)	Outcome	Intervention Group (Elective Induction at 39 weeks)	Comparator Group (Expectant Management)	Effect Estimate (95% CI)	p-value	Conclusion Regarding Outcome
Grobman et al., [5] (2018)	Composite perinatal death/severe complications	4.3%	5.4%	RR 0.80 (0.64–1.00)	Not reported	No significant reduction
	Cesarean delivery	18.6%	22.2%	RR 0.84 (0.76–0.93)	Not reported	Reduced cesarean rate
Elhoseny et al., [12] (2025)	Vaginal delivery rate	91.2%	70.6%	+20.6% higher	0.031	Elective induction significantly increases vaginal delivery rates
	Cesarean delivery rate	8.8%	29.4%	-20.6% lower	0.031	Elective induction reduces cesarean rates
	Maternal fever	23.5%	11.8%	Not reported	0.340	No significant difference
	Fetal distress, NICU admissions, Apgar scores, uterine hyperstimulation, significant maternal morbidity	No significant difference	No significant difference	Not reported	Not reported	No significant difference
Crawford et al., [13] (2025)	Perinatal mortality	aOR 0.48 (0.30–0.76)	Reference	0.002	Reduced perinatal mortality with induction	-
	Antepartum stillbirth	aOR 0.38 (0.15–0.97)	Reference	0.04	Reduced risk	-
	Intrapartum stillbirth	aOR 0.16 (0.04–0.66)	Reference	0.01	Reduced risk	-
	Severe neonatal neuro morbidity	aOR 0.46 (0.39–0.53)	Reference	<0.001	Reduced risk	-
	Severe neonatal non-neuro morbidity	aOR 0.65 (0.62–0.68)	Reference	<0.001	Reduced risk	-
	Severe maternal outcome	aOR 0.95 (0.92–0.99)	Reference	0.008	Slight reduction	-
	Maternal-infant separation	aOR 1.04 (1.00–1.08)	Reference	0.08	No significant difference	-
	Caesarean delivery	aOR 0.54 (0.51–0.58)	Reference	<0.001	Reduced risk	-
	Perineal trauma	aOR 0.53 (0.45–0.63)	Reference	<0.001	Reduced risk	-
	Shoulder dystocia	aOR 0.73 (0.64–0.84)	Reference	<0.001	Reduced risk	-
Zenzmaier et al., [14] (2021)	Cesarean delivery rate (i) vs next week or beyond	17.2%	16.2%	Adjusted OR 0.93 (0.86–1.00)	0.059	No significant difference
	Cesarean delivery rate (ii) vs at next week	17.2%	13.6%	Adjusted OR 0.76 (0.70–0.82)	<0.001	Significantly lower with expectant management (at next week)
Stock et al., [15] (2012)	Perinatal mortality	0.08%	0.18%	Adjusted OR 0.39 (0.24–0.63)	Significant (99% CI excludes 1)	Reduced mortality with induction
	Neonatal admission	8.0%	7.3%	Adjusted OR 1.14 (1.09–1.20)	Significant	Increased admissions with induction
Souter et al., [16] (2019)	Composite neonatal outcomes	No significant increase	No significant difference	Not significant	Not significant	Induction not associated with increased neonatal adverse outcomes
Sinkey et al., [17] (2019)	Perinatal composite morbidity	4.0%	7.1%	aOR 0.57 (0.34–0.96)	<0.05	Reduced morbidity with induction
	Cesarean delivery	5.1%	6.6%	aOR 0.60 (0.37–0.97)	<0.05	Reduced cesarean rate
	NICU admissions, other maternal outcomes	No significant difference	No significant difference	Not reported	>0.05	No significant difference
Park et al., [18] (2022)	Intra-amniotic infection	1.7%	3.0%	aRR 0.58 (0.49–0.68)	<0.001	Reduced risk with induction
	Blood transfusion	0.3%	0.5%	aRR 0.66 (0.45–0.98)	0.03	Reduced risk
	Low 5-min Apgar	0.31%	0.47%	aRR 0.66 (0.44–0.97)	0.031	Reduced risk
	Cesarean delivery	49.0%	27.6%	aRR 1.72 (1.68–1.77)	<0.001	Increased risk
	Other perinatal outcomes	No significant difference	No significant difference	Not reported	Not reported	No difference
Palatnik & Kominiarek, [19] (2020)	Macrosomia (parous women)	11.6%	17.6%	Adjusted OR 0.50 (0.38–0.66)	Not reported	Reduced risk with induction
Lee et al., [20] (2016)	Caesarean delivery (nulliparous)	Lower odds	Reference	OR 0.77 (0.63–0.95)	Not reported	Reduced cesarean risk
	Caesarean delivery (multiparous)	Lower odds	Reference	OR 0.67 (0.56–0.81)	Not reported	Reduced cesarean risk
	Macrosomia	Lower odds	Reference	OR not reported	Not reported	Reduced risk
	Other neonatal outcomes	No difference	Reference	Not reported	Not reported	No difference
Lappen et al., [21] (2015)	Failed TOLAC	45.6%	29.8%	Adjusted OR 2.16 (1.76–2.67)	Not reported	Increased risk with induction
	Composite neonatal morbidity	Not increased	Not increased	No significant association	Not reported	No difference
	NICU admission	Not increased	Not increased	No significant association	Not reported	No difference
Gibson et al., [22] (2014)	Cesarean delivery	23.6%	38.5%	Adjusted OR 0.47 (0.38–0.57)	Not reported	Reduced cesarean risk
	Neonatal morbidity	Lower (exact % not reported)	Higher	Adjusted OR 0.75 (0.61–0.92)	Not reported	Reduced morbidity
Pickens et al., [23] (2018)	NICU admission (nulliparous)	7.9%	10.1%	Adjusted OR 0.79 (0.70–0.89)	<0.05	Reduced risk
	NICU admission (parous)	5.3%	7.4%	Adjusted OR 0.75 (0.68–0.82)	<0.05	Reduced risk
Darney et al., [24] (2013)	Shoulder dystocia	Increased odds	Reference	OR not reported	Not reported	Increased risk
	Other neonatal outcomes	No difference	Reference	OR not reported	Not reported	No difference
Cheng et al., [25] (2012)	Caesarean delivery	35.2%	40.9%	Adjusted OR 1.25 (1.17–1.33)	Not reported	Reduced risk with induction
Bailit et al., [26] (2015)	Peripartum infections	Less frequent	More frequent	OR 0.66 (0.49–0.90)	Not reported	Reduced risk
	Severe lacerations	Less frequent	More frequent	OR 0.60 (0.42–0.86)	Not reported	Reduced risk
	Neonatal complications	Less frequent or no different	More frequent	Not reported	Not reported	Reduced or similar

Risk of Bias Assessment Results

The risk of bias assessment revealed that most included studies were of high quality, with nine cohort studies demonstrating low risk of bias [13-15, 18-20, 22-24, 26]. These studies utilized large, population-based datasets or multicenter cohorts with rigorous adjustment for confounders (e.g., maternal age, BMI, parity) and had complete follow-up, minimizing selection and comparability biases (Table 3). The sole randomized controlled trial [5] was also rated as low risk, with proper randomization, intention-to-treat analysis, and blinded outcome assessment (Table 4).

**Table 3:** Risk of Bias Assessment Using the Newcastle-Ottawa Scale (Cohort Studies).

Study	Selection (Max 4)	Comparability (Max 2)	Outcome (Max 3)	Total Score (Max 9)	Risk of Bias
Elhoseny et al., [12] (2025)	3	1	2	6	Moderate
Crawford et al., [13] (2025)	4	2	3	9	Low
Zenzmaier et al., [14] (2021)	3	2	2	7	Low
Stock et al., [15] (2012)	4	2	3	9	Low
Souter et al., [16] (2019)	3	1	2	6	Moderate
Sinkey et al., [17] (2019)	3	1	2	6	Moderate
Park et al., [18] (2022)	4	2	3	9	Low
Palatnik and Kominiarek, [19] (2020)	3	2	2	7	Low
Lee et al., [20] (2016)	3	2	2	7	Low
Lappen et al., [21] (2015)	3	1	2	6	Moderate
Gibson et al., [22] (2014)	3	2	2	7	Low
Pickens et al., [23] (2018)	3	2	2	7	Low
Darney et al., [24] (2013)	4	2	3	9	Low
Cheng et al., [25] (2012)	3	1	2	6	Moderate
Bailit et al., [26] (2015)	3	2	2	7	Low

**Table 4:** Risk of Bias Assessment for Randomized Controlled Trials Using the Cochrane RoB 2 Tool

Study	Randomization Process	Deviations from Intended Interventions	Missing Outcome Data	Measurement of Outcomes	Selection of Reported Results	Overall Risk of Bias
Grobman et al., [5] (2018)	Low risk (Computer-generated randomization, allocation concealment)	Low risk (Intention-to-treat analysis)	Low risk (<5% loss to follow-up)	Low risk (Blinded assessors)	Low risk (Pre-specified outcomes)	Low risk

Six studies had a moderate risk of bias [12, 16, 17, 21, 25], primarily due to smaller sample sizes, single-center designs, or limited adjustment for confounding variables. For example, Elhoseny et al. [12] and Sinkey et al. [17] relied on single-hospital data with partial control for maternal characteristics, while Lappen et al. [21] did not fully account for obstetric history in their TOLAC analysis. Despite these limitations, all studies clearly defined exposures and outcomes, and none were excluded for critical bias.

Discussion

The findings of this systematic review, which synthesized evidence from 16 studies on neonatal outcomes following eIOL at 39 weeks compared to expectant management, reveal a complex but largely favorable landscape. The most consistent observation across studies was the reduction in cesarean delivery rates with eIOL, as demonstrated by Elhoseny et al. [12], Gibson et al. [22], and Grobman et al. [5]. The mechanistic explanation for this reduction may lie in the avoidance of post-term complications, such as macrosomia or placental insufficiency, which increase the likelihood of cesarean delivery during expectant management [19, 25]. However, the increased cesarean risk observed by Park et al. [18] in women with prior cesarean deliveries suggests that the benefits of eIOL may not extend uniformly to all populations, underscoring the need for individualized decision-making.

Neonatal outcomes were generally reassuring, with several studies reporting reduced perinatal mortality and severe morbidity following eIOL at 39 weeks. Crawford et al. [13] documented a 52% reduction in perinatal mortality, while Stock et al. [15] found a 61% lower odds of perinatal death. These findings are supported by meta-analyses, such as those by Dahlen et al. [27], which concluded that induction at 39 weeks reduces stillbirth risk without increasing neonatal morbidity. However, the absence of a significant reduction in the composite adverse outcome in the Grobman et al. [5] RCT suggests that the mortality benefits may not always translate to measurable reductions in severe neonatal morbidity. This discrepancy could reflect differences in outcome definitions or study power, as the RCT was designed to detect a 35% relative risk reduction, whereas large cohort studies like Crawford et al. [13] had the statistical power to identify smaller effects.

The review also highlighted population-specific nuances. For obese women, eIOL at 39 weeks was associated with reduced macrosomia [19, 20] and lower NICU admissions [23], likely due to the avoidance of prolonged exposure to hyperglycemic intrauterine environments. In contrast, women with prior cesarean deliveries faced higher failed TOLAC rates with induction [21], though without increased neonatal morbidity. These findings align with ACOG guidelines, which recommend shared decision-making for eIOL in obese women but caution against non-medically indicated induction in TOLAC candidates [28]. The variability in outcomes across subgroups underscores the importance of tailoring induction strategies to maternal characteristics rather than adopting a one-size-fits-all approach.

Maternal outcomes were another critical dimension of this review. The reduction in peripartum infections [26] and severe lacerations with eIOL may reflect shorter labor durations or fewer operative deliveries, as suggested by observational data [24]. However, the modest reduction in severe maternal outcomes (5%) reported by Crawford et al. [13] suggests that the maternal benefits of eIOL, while statistically significant, may be clinically marginal. This contrasts with the more pronounced neonatal benefits, reinforcing the primacy of fetal indications in the decision to induce at 39 weeks.

The risk of bias assessment bolstered confidence in these findings, as the majority of studies (10/16) were rated as low risk. High-quality cohort studies like Crawford et al. [13] and Stock et al. [15] employed robust confounder adjustment, while the Grobman et al. [5] RCT minimized bias through randomization and blinding. However, the moderate-risk studies [12, 17] remind us of the limitations inherent in observational designs, particularly residual confounding by unmeasured variables like clinician preference or hospital protocols.

When contextualized within the broader literature, this review’s findings support a growing consensus that eIOL at 39 weeks is a safe and often beneficial option for low-risk pregnancies. A 2023 meta-analysis by Hong et al. [29] echoed our conclusion that eIOL reduces cesarean rates without compromising neonatal outcomes, though it emphasized the need for further research in high-risk subgroups. Similarly, the World Health Organization’s 2021 guidelines on labor induction cite evidence from studies like Grobman et al. [5] to recommend shared decision-making for eIOL in settings with adequate monitoring capacity. However, the increased cesarean risk in certain populations [18] and the marginal maternal benefits suggest that eIOL should not be universally mandated but rather offered as one of several management options.

Limitations

This review has several limitations. First, the predominance of retrospective cohort studies introduces the potential for residual confounding, despite adjustments in high-quality studies. Second, heterogeneity in outcome definitions (e.g., "expectant management" defined variably by Zenzmaier et al. [14]) complicates cross-study comparisons. Third, the exclusion of non-English studies may have omitted relevant data. Finally, the lack of long-term neonatal follow-up in most studies precludes assessment of outcomes like neurodevelopmental delay.

## Conclusions

Elective IOL at 39 weeks gestation is associated with significant benefits, including reduced rates of cesarean delivery and improved perinatal outcomes, without increasing neonatal morbidity in low-risk populations. The observed heterogeneity in outcomes-particularly among high-risk subgroups like women with prior cesarean deliveries or obesity-underscores the importance of individualized clinical decision-making. While eIOL at 39 weeks appears to be a viable strategy for mitigating adverse birth outcomes, its implementation should be guided by patient-specific factors, shared decision-making, and institutional resources to optimize both maternal and neonatal well-being.

Moving forward, future research should prioritize randomized controlled trials in underrepresented populations, such as those with gestational diabetes or fetal growth restriction, to clarify the risks and benefits in these cohorts. Additionally, long-term follow-up studies are needed to assess neurodevelopmental and childhood health outcomes following eIOL, as existing evidence primarily focuses on short-term perinatal metrics. By addressing these gaps, the medical community can refine clinical guidelines and ensure that the practice of elective induction at 39 weeks is both evidence-based and patient-centered, balancing the imperative to reduce preventable adverse outcomes with the need to avoid unnecessary interventions.

## Appendices

The search strategy used to retrieve articles is described in Table 5. 

**Table 5:** Database Search Strategy

Database	Search String
PubMed	("Induced Labor"[Mesh] OR "Labor, Induced"[tiab] OR "Elective Induction"[tiab] OR "Labor induction"[tiab] OR "Induction of labor"[tiab]) AND ("Gestational Age"[Mesh] OR "39 weeks"[tiab] OR "thirty nine weeks"[tiab] OR "39-week"[tiab]) AND ("Infant, Newborn"[Mesh] OR "Neonatal outcome"[tiab] OR "Neonatal outcomes"[tiab] OR "Neonatal morbidity"[tiab] OR "Neonatal mortality"[tiab] OR "Perinatal outcome"[tiab] OR "Newborn outcomes"[tiab])
Scopus	(TITLE-ABS-KEY("elective induction" OR "labor induction" OR "induced labor" OR "induction of labor") AND TITLE-ABS-KEY("39 weeks" OR "thirty nine weeks" OR "39-week") AND TITLE-ABS-KEY("neonatal outcome" OR "neonatal outcomes" OR "neonatal morbidity" OR "neonatal mortality" OR "perinatal outcome" OR "newborn outcome" OR "newborn outcomes"))
Web of Science	TS=("elective induction" OR "labor induction" OR "induced labor" OR "induction of labor") AND TS=("39 weeks" OR "thirty nine weeks" OR "39-week") AND TS=("neonatal outcome" OR "neonatal outcomes" OR "neonatal morbidity" OR "neonatal mortality" OR "perinatal outcome" OR "newborn outcome" OR "newborn outcomes")
Embase	('induced labor'/exp OR 'elective induction':ti,ab OR 'labor induction':ti,ab OR 'induction of labor':ti,ab) AND ('gestational age'/exp OR '39 weeks':ti,ab OR 'thirty nine weeks':ti,ab OR '39-week':ti,ab) AND ('newborn'/exp OR 'neonatal outcome':ti,ab OR 'neonatal outcomes':ti,ab OR 'neonatal morbidity':ti,ab OR 'neonatal mortality':ti,ab OR 'perinatal outcome':ti,ab OR 'newborn outcome':ti,ab OR 'newborn outcomes':ti,ab)
